# The Influence of Small Amounts of the Biobased Polyester PEF on the Mechanical Recycling of PET

**DOI:** 10.3390/polym18060668

**Published:** 2026-03-10

**Authors:** Roy H. A. Visser, Ele L. de Boer, Matheus Adrianus Dam, Ingrid Goumans, Robert Siegl, Ed de Jong

**Affiliations:** 1 Avantium Renewable Polymers BV, Zekeringstraat 29, 1014 BV Amsterdam, The Netherlands; 2 Alpla Werke Alwin Lehner GmbH & Co., KG, Mockenstraße 34, 6971 Hard, Austria

**Keywords:** recycled PET, mechanical recycling, bottle-to-bottle recycling, PEF, multilayer bottles, crystallization retardation

## Abstract

Reducing dependence on fossil-based feedstocks for packaging can be achieved through three complementary strategies: minimizing packaging use, increasing closed-loop recycling rates, and expanding the adoption of renewable (e.g., biobased) packaging materials. To ensure these defossilization pathways reinforce rather than hinder one another, it is essential to understand how new biobased materials interact with existing recycling streams. With the market introduction of packaging containing the biobased polyester poly(ethylene 2,5-furandicarboxylate) (PEF) approaching, several studies have investigated blends and copolyesters of poly(ethylene terephthalate) (PET) and PEF. This study expands current knowledge of thermomechanical and crystallization behavior by examining the influence of PEF on the mechanical recycling process of bottle-grade PET. Processing behavior was assessed at various PEF contents at both laboratory and industrial scales, and the resulting recycled resin and bottles were analyzed for color, crystallization behavior, and bottle performance. Although the melting temperature decreased with rising PEF content, no negative impact on the industrial recycling process investigated was observed for PEF levels up to 10 wt%. Two notable trends emerged: increasing PEF content reduced crystallization rate, yielding bottles with higher transparency, while yellowness also increased. Ongoing research aims to understand and mitigate this rise in yellowness.

## 1. Introduction

The recycling of plastics is crucial for Europe, as it reduces the industry’s dependency on fossil resources, lowers greenhouse gas emissions, and supports the transition to a circular economy, aligned with the EU’s sustainability and climate goals. As one of the most widely used plastics, PET (poly(ethylene terephthalate)) has a high potential for reuse, and its favorable properties related to recycling support the EU’s goals for a circular economy and sustainable materials management [1,2,3,4]. In 2022, approximately 5 million tonnes of PET packaging (including bottles, trays, and flexible packaging) were placed on the market in the EU27+3 region (EU member states plus Norway, Switzerland, and the UK). Of this amount, 3 million tonnes were collected and 1.4 million tonnes extruded in recycled PET (rPET) pellets [5]. These rPET granules mainly end up in bottles, leading to an average recycled content in European PET beverage bottles of 24%. While Europe is making strong progress toward circularity in PET use, disparities between countries and infrastructure limitations still pose challenges to meet future EU targets, such as the 30% recycled content mandate for beverage, food and cosmetics by 2030. A particular threat to reaching these targets is the challenge European recyclers face in competing with virgin and recycled PET prices from suppliers outside of the EU [6,7].

With the push for a higher quantity of rPET in the bottle market, keeping the quality of the resulting material pool up to par becomes both more challenging and important. The state-of-the-art in mechanical recycling of PET involves a multi-step process that includes collection, sorting [8], washing, drying, extrusion, and solid-state polycondensation (SSP). The SSP step is critical for restoring the molecular weight (and therewith the mechanical properties for high-performance applications), as well as to decontaminate the PET from volatiles not yet extracted during the (vacuum) extrusion step, which is critical to obtain food contact approved rPET [4,9]. As an alternative to the SSP step, innovative liquid state polycondensation processes are currently implemented, with the same goals to decontaminate and lift the molecular weight of the PET resin. Despite technological advancements, challenges persist in PET recycling, including contamination from and build-up of other, non-compatible, polymers (e.g., polyamides, PVC, EVOH, PLA) [3,10,11], non-intentionally added substances (NIAS) [12], chain scission during thermal cycles [4], color build up [9,13,14] and variability in feedstock composition [15], all of which can degrade rPET quality and final products made thereof [4,9,12,13]. With the drive and development towards higher recycling rates and recycled content in final articles, each of the items listed will put the quality of the rPET material pool under further pressure [16].

Increasing recycling rates will not meet the targets set to reduce the industry’s dependency on fossil feedstock alone [17]: overall plastic reduction (for example via reuse) and a move towards plastics produced from bio-based feedstock will also be required. The first bio-based polymer introduced commercially in the packaging market was PLA [18]. The low barrier performance and the negative impact of PLA on the recycling of PET [10] have limited the growth of PLA in the food and beverage bottle market. Recently, another bio-based polyester has attracted significant scientific and industrial attention as a packaging material candidate: poly(ethylene 2,5-furandicarboxylate) (PEF) [1,19,20,21]. PEF can be polymerized via polycondensation of 2,5-furandicaboxylic acid (FDCA) and ethylene glycol (EG, also referred to as monoethylene glycol, MEG in industry). Where bio-based EG is already commercially available, multiple companies are working on the market introduction of sugar-based FDCA. PEF itself is a polyester that has a close resemblance to PET with respect to its chemical structure. Consequently, PEF behaves in a similar fashion with respect to processing: existing PET processing equipment can be applied to convert PEF into end products while only modifying processing conditions. This has been demonstrated for injection stretch blow molding of containers [1], cast and biaxial stretched film production [22,23,24], as well as fiber spinning [25,26,27]. A key aspect for these end products is that PEF, like PET, is able to form strain-induced crystals upon stretching (uni- or biaxially) just above its glass transition temperature [28,29,30,31], which boosts the end-product performance while maintaining the clarity of the bottles.

**Figure 1:**
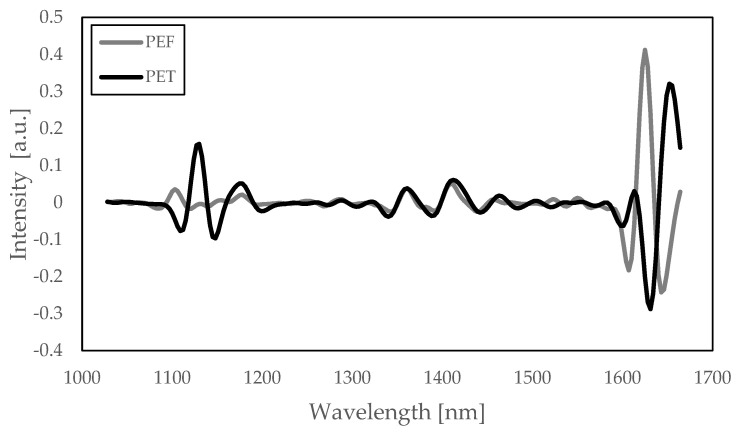
NIR spectrum of PEF in grey (intensity vs. wavelength in nm), where a PET spectrum is included as a comparison in black. Reproduced from [38], Research Centre NTCP, 2023.

One of the key benefits of PEF is its excellent barrier against gases [32,33,34,35,36,37]: a strong value in packaging materials. When compared to (amorphous) PET, the permeation rate of O_2_ through (amorphous) PEF is about 11× lower [32], of CO_2_ about 15× lower [19] and of water vapor about 2.8× lower [33]. With such barrier performance, food and beverage packaging are obvious end applications where the benefits of PEF can result in favorable business cases, either as a barrier layer in PET packaging or as a full PEF packaging solution (replacing, for example, glass). When targeting such applications, it is likely that PEF can end up in the PET recycling stream, for example, due to sorting errors. Research Centre NTCP (Heerenveen, The Netherlands) has carried out a sorting study of mono- and multilayer PEF bottles and trays, which was recently published online [38]. Despite the chemical similarities between PEF and PET, PEF has a distinctly different near-infrared (NIR) spectrum than PET (see Figure 1) and other common polymers in plastic waste streams. The report concludes that if the NIR spectrum of PEF is added to a sorting program, it allows correct sorting of PEF articles and does not lead to additional miss-sorting of HDPE and PP rigid items (into the PET stream). When separating the PET bottles from the deposit stream, including PEF bottles/trays, up to 2% of the PEF monolayer packages ended up in the PET stream. A multilayer PET bottle with a 10 wt% PEF middle layer was identified as a PET bottle, as the outer PET layer is thicker than the penetration depth of the reflected NIR signal. Consequently, both the market introduction of monolayer PEF bottles and the use of PEF as a barrier layer in PET will lead to some PEF entering the PET recycling stream. Extensive knowledge on the influence of low PEF content levels on the mechanical recycling process of PET and the performance of the resulting recycled resin is therefore of key importance to protect the quality and value of the recycled PET material pool.

During the mechanical recycling process of PET, there is always a melt processing step in which any remaining non-PET polymers are blended into the PET melt. Contrary to other (barrier) materials, PEF still has reactive end groups, which can transesterify with the PET during melt processing [39,40]. Several publications have reported on the behavior and properties of the resulting PET/PEF copolyester, for example, on miscibility [39,41], crystallization [39,42,43,44,45,46], thermal stability [42,44,46,47], barrier [43,44], visual appearance [44], and thermal/mechanical performance [43,44,47]. The results of these studies already provide good insights into what will happen when PEF-containing packaging enters the PET recycling process and into the properties of the recycled resin. It should be noted that these studies all have a homopolyester of PET as a reference and starting point for the PET/PEF blend, whereas the PET recycling feedstock consists of the majority of bottle-grade PET. Typically, ~2 mol% isophthalic acid (IPA) is added as a comonomer to bottle-grade PET to modify the crystallization kinetics to better fit the blow molding process. Therefore, it is of interest to investigate the properties of copolyesters with TPA, IPA, FDCA, and EG as comonomers. Moreover, to the best of the authors’ knowledge, no scientific studies have been published specifically on the influence of PEF on the mechanical recycling process of PET. Consequently, factual discussions on this topic between the various stakeholders in the recycling chain are challenging. In the meantime, several lab-scale recycling assessments have been carried out, which were the basis for several (interim) endorsements on the recycling of PEF-based containers in the PET recycling stream, such as an interim endorsement for monolayer PEF bottles up to 2% market penetration by the European PET Bottle Platform (EPBP) [48]. For PET bottles with up to a 10 wt% PEF barrier layer, several approvals have been granted worldwide: a RecyClass Technology Approval (EU) [49], Critical Guidance Recognition by the Association of Plastic Recyclers (APR, US) [50], recycling approval by Council for PET Bottle Recycling (CPBR, JP) [51] and an interim endorsement up to 5% European PET bottle market penetration by EPBP (EU) [52,53]. Each of these assessments has been conducted upon request by Avantium, using their PEF grades in combination with commercial bottle-grade PET. These results are put in perspective with existing literature data and provide further insights into the effects the market can anticipate when PEF end-products enter the market.

In this work, the procedure and results of the lab-scale assessments, which served as a basis for the interim EPBP and RecyClass endorsements, will be presented and complemented with a pilot scale study on the transesterification of PET/PEF blends, a commercial-scale recycling validation, including end product (containers) performance evaluation and a comparison with existing literature data on PET/PEF blends and copolyesters.

## 2. Materials and Methods

### 2.1. Materials

All PEF used in the research conducted for this paper was supplied by Avantium Renewable Polymers, Amsterdam, The Netherlands. Unless stated otherwise, the grade Releaf^®^ PEF RP90N with an intrinsic viscosity of 0.86 dL/g was used. Several commercial grades of PET from different suppliers were used: RAMAPET N1S (IV: 0.82 ± 0.02 dL/g) and PREFORMANCE^®^ 1708CC (IV 0.80 ± 0.02 dL/g), both from Indorama Ventures (Bangkok, Thailand), Laser+^®^ C (E60A) (IV 0.81 ± 0.02 dL/g) from DAK Americas LLC, a bottle grade PET supplied by a undisclosed Dutch resin supplier and M&G Cleartuf P82, a bottle grade PET from Gruppo Mossi & Ghisolfi (Toronta, Italy). All PET grades used in this study are bottle grades, and hence, all contain a certain amount (typically ~2 mol%) of IPA as comonomer.

### 2.2. Methods

#### 2.2.1. Transesterification Screening

The high-level transesterification screening was conducted on a MAS 24, a co-rotating, conical twin screw extruder with a final diameter of 24 mm from MAS Maschinen- und Anlagenbau Schulz GmbH, Pucking, Austria. As input material, a bottle grade PET from a Dutch supplier and PEF grade G90P with an IV of 0.88 dL/g from Avantium Renewable Polymers was used. The PET was dried for 16 h at 150 °C with dry air (dewpoint < −30 °C). The PEF was dried for 14 h at 140 °C and subsequently boost-dried for 4 h at 160 °C using nitrogen. For each experiment, a total of 6 kg of dry-blend was prepared by adding the right amount of PEF pellets to PET pellets to reach the targeted composition. The first 2 kg produced at each setting was discharged as flush material.

Additional experiments were conducted with the same PET and PEF resin grades, but on a co-rotating twin screw extruder. Prior to processing, the resins were dried in a vacuum oven between 140 °C and 150 °C.

^1^H-NMR was used to determine the PEF fraction in the blends, using a Bruker Avance III HD 600 operating (Bruker, Billerica, MA, USA)at 600 MHz. The sample is prepared by dissolving 10 mg of the PET/PEF blend in 0.7 mL 1,1,2,2-tetrachloroethane-d2 and subsequently measured at 23 °C for 32 scans. The fraction of PEF in the blend is determined from the ratio of the proton signals of the Furan ring in PEF (7.22–7.34 ppm, 2 protons) and proton signals of the Benzene ring in PET (8.04–8.21 ppm, 4 protons).

^13^C-NMR was used to determine the average block length of PEF fragments in the PET chain as a measure of the extent of transesterification, using a Bruker Avance II 500 operating at 125 MHz. The sample was prepared by dissolving 10 mg of the PET/PEF blend in 1 mL of a mixture of 95% trichloromethane and 5% trifluoroacetic acid and measured at 23 °C for 2048 scans. In the same way as the terephthalic acid in PET [54], the FDCA *ipso*-carbon at 146.3 ppm shifts slightly depending on whether the moiety on the other side of the ethylene glycol link is another FDCA (146.25 ppm) as in a connection between two PEFs (FEF), or alternatively, a TPA moiety (146.32 ppm) coming from a PEF–PET connection (TEF). The average block length of a PEF fragment in the PET chain was calculated as ([FEF] + [TEF])/[TEF].

DSC measurements were used for determining the thermal properties of PEF samples. First, 5 g of polymer is ground in a hand-held grinder, using a small amount of liquid nitrogen in the grinding cup to minimize the temperature increase that could occur because of shear heating during grinding. Then, a 5 mg sample was accurately weighed in a 40 mL aluminum crucible and sealed with an aluminum piercing lid. DSC analysis was performed on a DSC 1 STAR^e^ system of Mettler Toledo (Columbus, OH, USA), using Mettler Toledo STAR^e^ Software Version 15.00a. Temperature and heat flow were calibrated with a high-purity indium sample using standard procedures. Temperature scans were conducted under nitrogen flow (50 mL/min) according to:

- Heating ramp from 25 °C to 300 °C at 10 °C/min;
- Isothermal conditioning at 300 °C for 3 min;
- Cooling ramp from 300 °C to 25 °C at 10 °C/min;
- Isothermal conditioning at 25 °C for 3 min;
- Heating ramp from 25 °C to 300 °C at 10 °C/min.

The temperature at the peak value of the endotherm corresponding to the melting of the crystals is reported as the melting temperature, *T*_m_. The first heating ramp was used for the determination of *T*_m_ to minimize the influence of further transesterification during the measurement itself. The midpoint temperature in the glass transition region is reported as the glass transition temperature, *T*_g_, and was determined from the second heating ramp, as poor contact between the specimen and the DSC crucible disturbs the low-temperature range of the first heating ramp. The crystallization enthalpy, Δ*H*_c_, was determined from the area of the exothermal crystallization peak during the cooling ramp. All of the reported values related to DSC measurements were determined from a single DSC measurement per specimen.

#### 2.2.2. Lab-Scale Evaluation of PEF in PET Recycling

**Figure 2:**
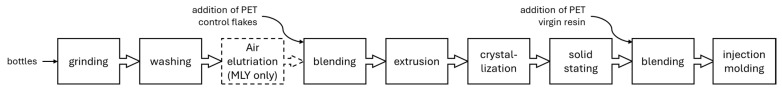
Flowchart of the steps incorporated in the EPBP route 1 assessment.

The influence of bottle innovations was assessed according to the EPBP protocol for three routes: (1) bottle-to-plaque, (2) bottle-to-bottle and (3) bottle-to-fiber. The first two routes were performed by the European laboratory of Plastic Technologies Incorporated (PTI) in Yverdon-Les-Bains, Centexbel in Gent Zwijnaarde Belgium performed the third route (not included in this study). Route 1 bottle-to-plaque incorporates the steps as schematically displayed in Figure 2.

Two separate route 1 assessments have been performed: one for monolayer PEF bottles and one for multilayer PET bottles with a 10 wt% PEF layer. The monolayer PEF bottles used originated from two different development batches, resulting in a blend with 50% bottles with an IV of 0.81 dL/g and 50% with an IV of 0.85 dL/g (IV measurement method described below). The reference bottles were produced with RAMAPET N1S. For the multilayer bottle assessment, both the reference bottles and the multilayer bottles were made using the PET grade PREFORMANCE^®^ 1708CC. For the barrier layer, the PEF grade X0573 (IV: 0.86 dL/g) was used. The preform injection was performed on a HyPET 120 preform injection molding machine from Husky Injection Molding Systems Ltd. (Bolton, ON, Canada), with a Ø38 mm screw for the main unit and Ø10 mm for the b-layer. The melt temperature in the main injection screw was kept at 280 °C and at 250 °C in the barrier layer screw. A 4 cavity, 19.8 g general-purpose carbonated soft drink preform mold was used. The PEF barrier was positioned with a closed dome and core biased (barrier layer more towards the inside of the bottle). The preforms were blown on a SBO 1 bottle blowing machine from Sidel Blowing & Services (Octeville-sur-Mer, France) into a 500 mL petaloid base bottle. The areal stretch ratio applied in the blowing process was 11.9. The reference preform was blown at 98 °C and the multilayer preform at 100 °C. Minor adjustments to the blowing parameters were made to end up with bottles with the same wall thickness distribution.

The grinding step was performed with a standard mechanical grinder with a 12 mm screen. The resulting flakes are washed using the standard European wash protocol (with flakes friction). Both the pre-wash (with caustic and detergent) and the main wash (with detergents only) were carried out at 85 °C. After the washing steps, the flakes were rinsed and dried. In the multilayer bottle assessment, an elutriation step was performed, where the flakes passed a wind-sifting device. Thinner flakes, for example, delaminated barrier layers, moved further along the air flow, and could, as such, be sorted from thicker flakes. The air speed in the elutriation setup was tuned such that a removal rate of 0.1 wt% was achieved for the control sample. This same air speed was used for the elutriation step of all samples.

For both the monolayer and the multilayer assessment, two blend ratios were studied, resulting in a total of 6 samples, which are listed in Table 1.

Prior to extrusion of the flakes, these were ground using a 4 mm screen to facilitate the feeding and avoid bridging of the flakes. The flakes were dried to a moisture level below 100 ppm in a minimal 3 h at 160 °C. Extrusion of the blends was conducted on an Arburg Allrounder 370 (single screw) injection molding machine (Arburg GmbH, Lossburg, Germany) converted into extrusion mode and equipped with a 40/250/40 mesh melt filter. The extrusion was carried out at 275 °C for all samples and at a screw speed of 10 rpm for the monolayer study samples and at 7 rpm for the multilayer study.

The pellets were dried and crystallized. The samples for the monolayer assessment were crystallized at 160 °C in a vacuum oven, and the multilayer samples were crystallized at 175 °C in a convection oven under a nitrogen sweep. The subsequent solid-state polymerization (SSP) step (also referred to as decontamination or post-condensation step) was performed at a reactor temperature of 205 °C (corresponding to a pellet temperature between 190 °C and 195 °C) under a nitrogen sweep for 8 h. The SSP time started once the pellets reached a temperature of 180 °C.

Prior to injection molding, each sample was dry blended with virgin PET granules of the respective reference grade at a 50/50 wt% ratio. Each resulting blend was injected in the shape of a 3 mm thick plaque using an Arburg Allrounder 370 and at a melt temperature of 275 °C and 15 °C mold temperature. The cycle time was kept at 32.1–32.2 s, resulting in a residence time in the range of 4–6 min.

Color measurements on flake and granule samples were conducted in reflectance using a Minolta CM-3600D (Konica Minolta Inc., Tokyo, Japan) and a HunterLab UltraScan VIS spectrophotometer (HunterLab, Reston, VA, USA) and are reported in CIELAB color space. Here, L* is a measure for the perceived lightness (a value of 100 being white and 0 being black). The value of a* represents how green or red a sample is, a value of 0 being neutral, positive values towards red and negative towards green. Similarly, b* is a measure for how blue or yellow a sample is, again, 0 being neutral, positive values towards yellow and negative values towards blue. The plaque samples were measured in transmittance using the same device, which also allowed for the determination of the haze. The amount of measurements varies per sample type and is reported in the footnotes of the respective table. The color of some pellets and molded samples was (also) measured in solution. Solution measurements allow comparison of the color irrespective of shape and crystallinity. The samples are dissolved in 1,1,1,3,3,3-Hexafluoro-2-propanol (HFIP) at a concentration of 30 mg/mL in a 40 mL glass vial (other diameter 27.5 mm). The absorbance was measured in total transmission mode for a wavelength range of 360–780 nm in steps of 10 nm. The absorbance at 400 nm is a measure for the yellowness of the sample and is normalized for any turbidity by subtracting the baseline absorbance as measured at 780 nm.

The intrinsic viscosity (IV) was determined in accordance with ASTM D4603. All reported values originate from a single measurement per sample. The IV lift rate is determined from a linear fit on the IV versus SSP time as measured after 0 h, 2 h, 4 h, 6 h and 8 h. The slope of the fit is reported. The acetaldehyde (AA) content according to ASTM F2013 after grinding the pellets to powder in liquid nitrogen.

Bottles were made from PEF containing rPET using a similar protocol as shown in Figure 2. Up to the SSP step, the same process as described above was followed using a starting point of a flakes blend with 2 wt% and 5 wt% of monolayer PEF flakes. Both the PEF and PET flakes originated from the same bottles as the bottle-to-plaque study, and SSP’ed resin with comparable properties was obtained in this bottle-to-bottle assessment. Instead of the injection molding of plaques, the SSP’ed granules are mixed with 50 wt% M&G Cleartuf P82 (a bottle grade PET from Gruppo Mossi & Ghisolfi) and injection molded into a 43 g preform (single cavity) using a melt temperature of 275° and a cycle time of 32 s. The preforms are blown into a 1.5 L straight wall bottle using a Sidel SBO1 blow molding machine. A total of 101 and 97 bottles were produced from the recycled granules, which originated from the 2 wt% PEF and 5 wt% PEF flake feedstocks, respectively. The latter bottles were heated with 1% more lamp power, resulting in a higher blowing temperature (113.5 °C versus 111 °C). The rest of the blowing parameters were the same for both samples.

The maximum internal pressure a bottle can withstand is determined by applying a pressure ramp until failure. Per sample, 12 bottles have been tested; both the maximum pressure and the bottle expansion (in volume) are reported.

The top load resistance was assessed by loading a (vented) unfilled bottle with an increasing force and recording the peak load as well as the elongation at this peak load. A total of 12 bottles were tested, and the mode of failure is recorded.

The drop tests were conducted by dropping a bottle from 180 cm height in a vertical and a horizontal position. For the 2 wt% sample, 10 bottles were tested, and for the 5 wt% sample, 8 bottles were tested. Two sets of drop tests are carried out: one at 4 °C and one at 22 °C. After each drop, the bottle is checked for failures, and the number of passes is recorded.

The CO_2_ retention testing was carried out on 10 carbonated bottles (to 4 CO_2_ volume) for which the CO_2_ concentration was measured using infrared over a 49-day period. The trend in CO_2_ was extrapolated to a 17.5% loss using a linear fit, resulting in an estimate of the CO_2_ shelf life, which is reported in weeks.

#### 2.2.3. Industrial Validation

In the industrial validation trial, virgin PEF granules were added to a mechanical recycling production line at scale at the end of a commercial rPET production run of a European recycler. The PEF was added to the PET flakes in the regranulation process at concentrations of 0 wt%, 2 wt%,5 wt% and 10 wt%. Per sample, >500 kg of recyclate was produced at standard commercial operating conditions for each step in the process. The SSP’ed resins were used to injection mold two types of preforms/bottles: (1) a 500 mL CSD and (2) an aerosol container. Both preforms were molded on a Husky HyPET 90 tonnes injection molding machine, and the bottles were blown on a Soplar ALS1-4 blow molding machine (Soplar SA, Altstätten, Switzerland). For both bottles, the standard vPET processing conditions were modified to allow good rPET preforms/bottles production. The modifications towards using the rPET resins with PEF content were minor.

## 3. Results

### 3.1. Transesterification of PEF into PET

A first screening was performed on the influence of melt processing conditions of blends of PET and PEF on the degree of transesterification. Only blends with PEF as minor components were included in this study. During the residence time in the extruder, the molten PEF and PET chains undergo transesterification reactions; PEF with PEF chains, PET with PET chains, but also PEF with PET chains (and vice versa). As a result, the PEF gets incorporated into the PET chains. It is expected that a higher residence time, a higher melt temperature, a lower PEF content, and more severe mixing all promote a more random distribution of the PEF repeating units in the PET chain. A first screening study has been performed on whether and how the first three variables mentioned above influence the block length, and in Table 2, the experimental setup as well as the resulting polymer analytics are listed. Samples A–E were processed in a conical co-rotating twin screw extruder with a throughput range of 8–16 kg/h, a melt temperature range of 278–295 °C and a PEF content ranging from 2–10 wt%. As the experimental goal was limited to scoping the variables of interest, only single-point experiments/measurements were performed.

The block length, as determined by ^13^C-NMR, is an indicator for the degree of randomness of the distribution of the repeating units of PEF in the PET chains. The lower this value, the more random the repeating units are distributed. The visual appearance of the amorphous pellets gives an indication of how much of the PEF was incorporated in the PET in combination with how randomly the PEF repeating units are distributed. As long as larger domains of PEF are present, the blend will appear hazy as PEF and PET are not compatible as a blend [39,41]. The melting point of the crystalline phase (*T*_m_) is an indicator of how much of the PEF has been built into the PET chains. PEF chains that have not been built in yet will not form any crystals during the heating ramp of the DSC measurement [55], whereby PEF, which has been built into PET, will reduce the melting point of the resulting copolyester compared to that of the PET control sample.

Comparing the analytics on sample A and B indicates that the temperature plays a role in randomizing the copolyester as both the block length reduces, and the pellets become clearer. In addition, the 2 °C reduction in melting point for sample B points to a higher PEF content, though this minor difference can also be an effect of randomization. The influence of residence time is displayed in the comparison between samples B and C. Where the degree of randomization seems comparable, both the visual appearance and *T*_m_ of sample C with the lower residence time indicate that less PEF has been incorporated into the PET chains than sample B, especially considering the *T*_m_ is hardly lower than sample D, where only 2 wt% PEF was used. The latter sample shows that less PEF content leads to a higher degree of randomization than reference sample B. Finally, sample E is a midpoint sample with both a higher throughput, lower melt temperature and lower PEF content than reference sample B. Both the randomization is low (high block length), and the amount of PEF incorporation is not complete (*T*_m_ as high as sample D, which has much lower PEF content). These five samples provide first insights that all three variables play a role in the transesterification process. A further high-level conclusion is that even at PEF content levels up to 10 wt% PEF can be incorporated with a high degree of randomization with only one (reactive) extrusion step.

In a separate study, the influence of the PEF content level on the *T*_m_ for samples with a high degree of randomization was examined. Here, the reactive extrusion was conducted on a conventional co-rotating twin screw extruder with a throughput and temperature to ensure low block length values, even for the sample with 30 wt% PEF. Where no clear trend was observed for the glass transition temperature, the melting point has a pronounced downward trend with increasing PEF content. In Section 4, the correlation is visualized along with other data points in this study.

These explorative results indicate that PEF should not raise issues with haze in the recyclability tests. The focus for the recyclability assessments will be on PEF content levels below 5 wt%, as the PEF is expected to enter the PET stream due to sorting errors or as a barrier layer in multilayer bottles. Therefore, the drop in melting point is expected to not exceed ~5 °C. Recycling tests have been carried out to what extent this drop in melting point influences the recycling process and the quality of rPET bottles.

### 3.2. Laboratory-Scale Assessment of Influence of PEF in Mechanical Recycling of PET

The laboratory-scale assessment consists of the main steps, which are also found on commercial-scale recycling. Whereas multiple mechanical recycling technologies are used in the market, the EPBP protocol, which was followed in this study, was developed to provide a realistic indication of the PET recycling behavior at scale. After each step in the laboratory-scale mechanical recycling assessment, the key properties of the resulting samples were determined. The first two steps in the process are grinding and washing the bottles into washed flakes. No marked differences between the samples were observed during the grinding or washing step. Neither the intrinsic viscosity nor the color changed significantly after washing for any of the samples, as can be seen in Table 3.

#### 3.2.1. Elutriation

During the elutriation step on the multilayer PET/PEF flakes, 3.9 wt% lighter flakes were removed, compared to 0.1 wt% for the PET control (which was on target). Compositional analysis of the flakes using ^1^H-NMR did not yield a consistent dataset. The flake samples are still inhomogeneous, complicating the preparation of a representative milligram-scale specimen for the ^1^H-NMR measurement, leading to a large variation. Therefore, the PEF content in the final plaques was measured and determined at 0.8 wt% for the multilayer sample at a test concentration of 25% and 1.8 wt% for the multilayer sample at a 50% test concentration. By multiplying the dilution factor (8 for 25% test concentration and 4 for 50% test concentration), the PEF content in the multilayer flakes after elutriation (but before blending) can be calculated. The resulting values of 6.4 wt% and 7.2 wt% are not identical, but quite close considering the accuracy of the ^1^H-NMR analytics. From these numbers, the PEF content in the elutriated fraction can be calculated to be 99 wt% and 79 wt%, respectively. Considering the sensitivity of both values to measurement errors, they should be seen as a rough indication only.

#### 3.2.2. Extrusion

No issues, such as flake sticking, fumes/odors or feeding issues or distinct differences in process conditions were observed during extrusion of all flake batches. After each batch, the melt filter was replaced and found to be free from residues in all cases. Except for the values of b*, no marked differences in IV level or color were found for the PEF-containing samples compared to the PET controls. The b* increases with increasing PEF content.

#### 3.2.3. Crystallization and SSP

The amorphous extruded pellets were crystallized prior to the SSP process to prevent pellet-to-pellet sticking from occurring. No difference in sticking behavior during the subsequent SSP step was observed between the batches. The SSP step has two main goals to achieve: (1) it removes (part of) the volatile contaminants in the polymer feedstock during the mechanical recycling process, and (2) it allows molecular weight build-up to compensate for the IV loss the material has faced during one complete life-cycle loop. For both aspects, the rate of IV increase is of importance. Too high an IV increase rate would reduce the SSP time, potentially leading to incomplete decontamination. Too low an IV increase would increase the required SSP time and therewith decrease the production capacity and increase operational costs of recyclers. The IV increase rate determined for the PEF containing samples and the reference PET values are listed in Table 3. Even for the sample with 5 wt% PEF (the sample with the highest PEF content), the IV increase rate remains within the allotted 10% difference compared to the control samples. The color measurements on the SSP’ed pellets show a lower value on both a* and b* and a significant increase in L*, which is caused by the crystallinity in the pellet. The trends of all color values as a function of the PEF content remain similar.

DSC measurements were performed on the SSP’ed pellets (and listed in Table 3) to quantify the influence of PEF on the glass transition temperature (*T*_g_) and the crystallization behavior of PET. The increases in glass transition temperatures are more prominent for the rPET samples, which contain monolayer PEF bottle flakes, compared to the increase for samples with multilayer flakes. The increase of the first is unexpectedly high at 3.7 °C and 3.9 °C for 2 wt% and 5 wt% PEF content, respectively. Considering a difference in *T*_g_ between PEF and PET is 12 °C [19] and the PEF concentration in each sample, one would expect a *T*_g_ increase of less than 1 °C for a random copolyester. The values for the cold crystallization peak (*T*_c_) as determined from the second heating ramp and listed in Table 3 clearly show an increasing trend with an increase in PEF content, indicating that the crystallization rate is reduced with higher PEF content. Also, the melting point of the crystals formed reduces with increasing PEF content levels, to a maximum reduction from 245 °C to 238 °C for the PEF sample with 5 wt% PEF. The formation and perfection of crystals play a key role in preventing pellet-to-pellet sticking during the SSP/decontamination step in the mechanical recycling of PET. Although the drop is smaller than the maximum allowed deviation from the control, which is set out to be 10 °C in the EPBP protocol, it is worth paying attention to the sticking phenomenon in actual recycling assets when high PEF content levels are present in the PET.

For some PET bottle applications, such as water bottles, the acetaldehyde (AA) content in the PET resin is of importance as it gives an off flavor in the water. Therefore, the AA content was determined in the SSP’ed pellets. If anything, the AA content shows a decreasing trend with an increase in the PEF content. Based on these values, no negative effects are expected within the range of PEF content levels examined within this study.

#### 3.2.4. Injection Molding of Plaques

The SSP’ed pellets were injected into 3 mm thick plaques with the main purpose of assessing the color for end-products from the recycled material. The color was measured in transmission, also allowing the determination of haze in the plaques. There are strict guidelines for the color values of the resulting plaques: L* > 87, a* < 3, Δb* < 1.5 (the difference in the b* of the PEF containing sample and the control PET sample) and haze < 8%. The values obtained are listed in Table 3, and both the 5 wt% PEF monolayer sample and the 50 wt% test concentration multilayer sample do not pass the Δb* criterion (maximum 1.5 difference in b* between the recycling from the test bottle versus that of the PET reference bottle). The L*, a* and haze values for all evaluated samples comply with the color requirements for the injection-molded plaques. As a result of not passing the Δb* criterion, the interim endorsements were based on the results for the 2 wt% monolayer sample and the 25 wt% multilayer sample.

#### 3.2.5. Bottle Blowing

Further evaluation was carried out on producing bottles from a new batch of rPET containing PEF, which was produced using the same protocol as above. Two blends with monolayer PEF flakes prepared with a PEF content level of 2 wt% and 5 wt%. The preform and bottle analytics and performance are listed in Table 4. No dedicated PET control preform or bottle was produced for this study; the values in the control column are values measured on PET bottles with same bottle geometry, which were still in stock. The preform/bottle IV and color values do not differ significantly, except for the b*. The 5 wt% has a clearly higher b* (more yellow) when compared to the 2 wt%. All bottles have an exceptionally low haze value, with the 5 wt% PEF bottle the highest and the 2 wt% PEF bottle the lowest haze.

The bottles were first assessed on the following parameters: bottle dimensions, material distribution, section weight, bottle capacity, and expansion upon pressurizing and storage at elevated temperature. All values were similar to the values of the typical PET bottle and passed the bottle requirements. On the mechanical bottle evaluations (burst, top load and drop test), the differences between the control and the two PEF-containing samples are marginal and, if anything, show a slight improvement for PEF-containing samples (burst pressure). For the CO_2_ shelf life, no data for the control samples were measured due to the limited availability of PET reference bottles. The 5 wt% PEF-containing sample shows a significant CO_2_ shelf-life improvement compared with the 2 wt% bottle, which is in line with expectations as PEF has a much better barrier against CO_2_ than PET [35]. Overall, the lab scale bottle assessment demonstrated that bottles produced from rPET with a PEF composition of 2 wt% and 5 wt% can pass all requirements set forward on bottles by the EPBP.

### 3.3. PEF in Mechanical Recycling of PET on Industrial Scale

The recycler reported no deviations from the standard process other than the observation that the 10 wt% sample contains yellow pellets (a minority compared to the whiter pellets). Other than that, the color of each sample (as measured in reflection on the pellets) was reported to be within the typical margin of color variation observed for different feedstock batches. The strands that contained PEF appeared clearer. Also, no issues were reported for the post-condensation/decontamination process and the four rPET samples were reported to have an IV of 0.84 ± 0.2 dL/g. The SSP’ed pellets were injection molded into 3 mm thick discs and preforms, which were converted into 500 mL bottles. The analytical results on these samples are listed in Table 5.

The PEF content, as determined using ^1^H-NMR gives higher values than the targeted values. For the 10 wt% SSP’ed pellets, sample a large pellet-to-pellet variation was measured as well as visually observed (pellet color variation). However, for two ^1^H-NMR measurements on the neck of the bottle, the difference in the determined PEF was only 0.1 wt% (and the average value with 10.8 wt% close to the targeted value). No further polymer analyses on the SSP’ed pellets are reported here to avoid disturbances in the results due to pellet-to-pellet variation.

#### 3.3.1. Color Validation on Discs

**Figure 3:**
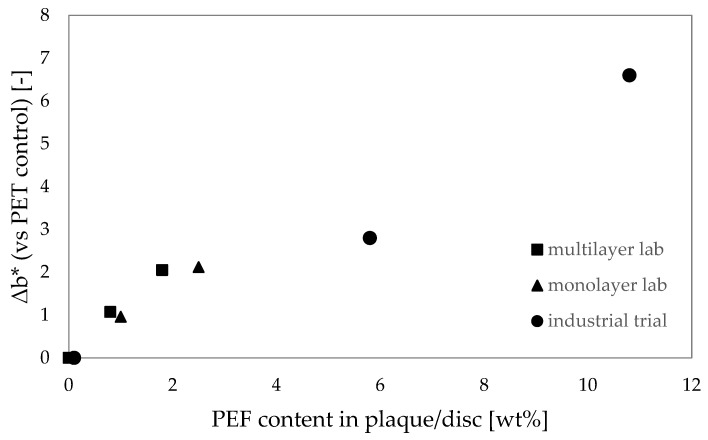
The increase in b* for PEF containing 3 mm injection molded plaques and discs when compared to the full PET reference, as a function of the PEF concentration.

The four rPET samples have been injection molded into 3 mm thick discs to allow a comparison of the discoloration for the PEF-containing samples with the full rPET reference, in line with the procedure for the lab scale assessment. The resulting L*,a*, b* and haze values as measured on three discs are reported in Table 5. For these rPET batches, the L* is already low for the reference rPET and further increases with the PEF concentration. The a* value increases with PEF concentration (towards red), but not as significantly as the increase in b*. Again, PEF pushes the recycled PET more towards yellow in the color spectrum, except for the sample with 2 wt% PEF: here, a drop of 0.2 in b* was observed. No explanation was found for the lower value; however, a decrease in b* has been observed before with low PEF levels in bottles (unpublished results). In Figure 3 the Δb* found in the lab scale assessments is shown next to the results found in the industrial validation trial. For the commercial recycling technology applied in this study, the lab-scale assessment as performed according to the EPBP protocol provides a good worst-case indication for the discoloration which is to be expected at the moment PEF indeed enters the PET recycling stream.

The haze in the plaques shows a decreasing trend with increasing PEF content. This effect is not in line with the results of the lab scale assessment as presented in Table 5. Different than for the lab scale assessment, the PET feedstock used for the industrial trial is much more diverse and partially will have seen multiple recycling loops. Therefore, the contamination profile is different, which will affect the optical [13] and crystallization behavior of the rPET [58] itself. Indeed, for the rPET control sample in the industrial validation, a value of 37.5 J/g was measured for the Δ*H*_c_, which is significantly higher than the 30.8 J/g which was measured on the rPET control sample in the lab scale multilayer assessment. Consequently, part of the haze in the industrial-scale rPET control sample could originate from crystals that have formed during the cooling process in the injection molding cycle. The addition of 2 wt% PEF already reduces the Δ*H*_c_ to 30.1 J/g, so comparable to the crystallization speed of the rPET control in the multilayer lab scale study and reducing the chance of crystal and thus haze formation.

#### 3.3.2. Bottle Validation

**Figure 4:**
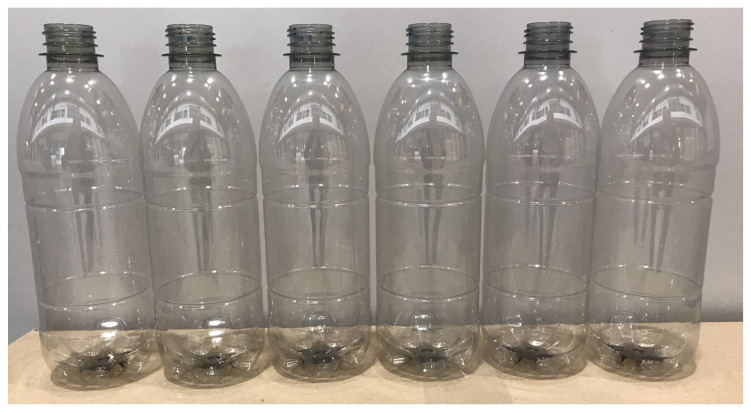
Visual appearance of the 500 mL bottles produced from the rPET resins produced in a commercial recycling process. From left to right, the bottles are produced from rPET with 10 wt% PEF, 5 wt% PEF, 2 wt% PEF, 0 wt% PEF (reference), 5 wt% PEF + toner and 10 wt% PEF + toner.

The color of the produced bottles was analyzed in transmission mode and on the label area of the bottle. The b* of the reference full rPET bottle was higher than the PEF-containing bottles, which is surprising, as for the molded plaques from the same resins the b* increased with increasing PEF content. Also, the L* is significantly lower for the full rPET reference bottle, meaning the bottle wall is darker and more yellow than the bottles with PEF content. For the PEF-containing bottles, the b* does show an increase with increasing PEF content, which is in line with the results of the lab-scale assessment. Two additional bottles were made with 5 wt% PEF and 10 wt% PEF with the addition of a commercially available rPET anti-yellow toner masterbatch. For both toned bottles, the b* could be reduced to a b* value of 1.6 at the expense of a reduction of the L* to 93.0 and 92.9, respectively. The commercial anti-yellow toner masterbatch thus shows itself to be capable of counteracting the discoloration caused by the addition of PEF. Visually, the 2 wt% bottles, as well as the toned bottles, are difficult to distinguish from the reference bottle (see Figure 4).

As explained above, the crystallization behavior of the recycled resins was explored with DSC measurements on specimens taken from the neck of the bottles. As the bottle necks have undergone two melt processing steps, the PEF is assumed to be highly randomly distributed (low block length). In line with the results reported in Section 3.1, a decrease in *T*_m_ was measured with the increase in PEF concentration due to the influence PEF has on the crystalline structure of the resulting copolyester. In addition, the rate of crystallization goes down, as demonstrated by the decrease in Δ*H*_c_; less crystals are formed upon cooling with higher PEF content levels. The crystallization retardation effect of the PEF, which is incorporated in the PET chains as observed with the decrease in Δ*H*_c_, is likely the reason for the reduction in haze for both the 3 mm discs and the bottles with increasing PEF content levels. The larger difference in haze for the bottle can also (partially) be caused by the relatively high areal stretch ratio for the preform-bottle combination used. This could have led to overstretching for PET, whereas it is known that PEF has a higher natural draw ratio [22] and forms crystals at higher strains [59]; possibly reducing the chance for overstretching of the PEF-containing preforms. In the next subsection, the recycled PET samples will be used in thick-walled bottles with a low areal stretch ratio to explore the potential of the retardation effect.

The crystallinity, as determined with a DSC analysis on the bottle wall of each sample, is listed in Table 5. The crystallinity is determined from the (endothermic) melting enthalpy of the first heating ramp and provides an indication of the amount of strain-induced crystals that have formed during the stretching step in the bottle blowing process. Where the ~75% drop in Δ*H*_c_ of the sample with 10 wt% PEF points at a significantly slower quiescent crystallization rate, the crystallinity in the bottle wall suggests the strain-induced crystallization rate is not as significantly affected by the presence of PEF: only a ~13% drop for the bottle with 10 wt% PEF.

The mechanical performance of the bottles (top load and burst pressure in Table 5) are mainly governed by the wall thickness distribution of the resulting bottle. The bottle with 2 wt% PEF content was blown with the same blowing parameters as the rPET reference bottle, resulting in a slightly different wall thickness distribution. For the bottle with 5 wt% PEF the blowing process parameters were slightly adjusted to achieve the same wall thickness distribution. Both the top load and the burst pressure of the bottles with 5 wt% are identical to the full rPET reference. This demonstrates that the bottle performance does not have to go down for rPET with up to 5 wt% PEF.

#### 3.3.3. Crystallization Retardation in Thick-Walled Bottles

**Figure 5:**
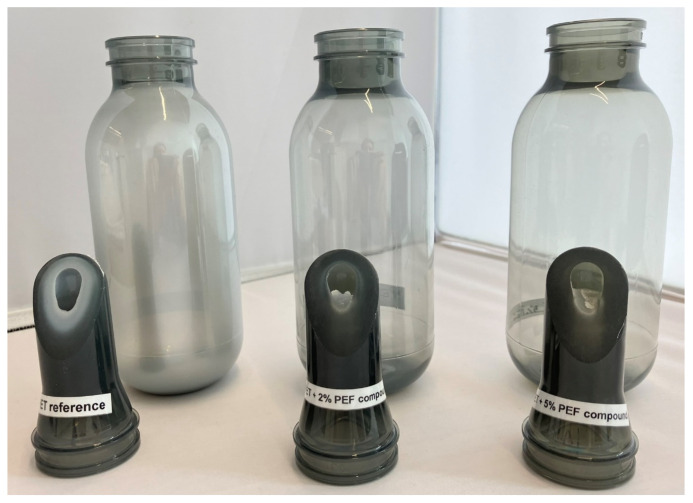
The preforms and bottles produced from full rPET, rPET + 2 wt% PEF and 5 wt% PEF (from left to right). The preforms were cut open at a 45°angle from the longitudinal axis, and the surface was polished to visualize the haze/crystallization through the thickness of the preform wall.

It is known that the crystallization speed is influenced by the recycled content as rPET typically has a higher crystallization rate than virgin PET [13]. For bottle producers, this can become an issue when striving towards higher recycled content levels as is dictated by, for example, the European Packaging & Packaging Waste Regulation legislation. Especially for thick-walled bottles, such as reusable bottles or aerosol containers, this can lead to haze formation. This issue was demonstrated by producing aerosol containers and the corresponding preforms from the full rPET reference and the rPET containing 2 wt% and 5 wt% of PEF. The preform used had a maximum thickness of 6.8 mm, leading to long cooling times in the injection molding process. Consequently, the reference preforms had a large amount of haze at the core of the preform (a white appearance) as shown in Figure 5. The preform could still be used to produce a bottle, but the bottle had a strong haze. For the preform made with rPET containing 2 wt% there was still a light hazy halo around the core of the preform, whereas this halo was completely removed for the rPET containing 5 wt% PEF. These results indicate PEF can play the role of clarifying agent for rPET, like the role isophthalic acid (IPA) has for virgin PET. In Section 3.1 it was concluded that for moderate PEF concentrations, it is possible to incorporate PEF in the PET chain and randomly distribute the FDCA moieties. In a typical recycling loop for PET bottles, the PET undergoes two melt processing steps; one during the regranulation of flakes and one during the preform production from granules. As demonstrated here, adding PEF in the PET recycling process can be used to tune the crystallization kinetics of PET.

## 4. Discussion

### 4.1. Transesterification

The transesterification reactions taking place during the melt processing (and thus recycling) of PET with PEF play a key role in the performance of the resulting resin. Riaz et al. [40] recently published data on the influence of processing temperature on the resulting resin performance and the bottles made thereof. In line with the results presented in Section 3.1, Riaz et al. report a strong correlation between the processing temperature within the (twin screw) extruder and the degree of randomness of the resulting copolyester [39]. The higher the melt temperature during the 40–50 s residence time in the extruder, the higher the degree of randomness, the lower the haze and the lower the gas barrier of amorphous films. The influence of the degree of mixing during melt processing has not been published in a single study for reactive blending of PEF in PET. In the study of Papageorgiou et al. [39], it took 7.5 min to get a single glass transition temperature for a 60/40 PET/PEF ratio in a DSC pan (so in the absence of shear mixing) at 280 °C. This significantly higher time required to reach at least some amount of randomization, can be caused by both the difference in the degree of mixing and the higher PEF content than that of the study of Riaz et al. A more detailed transesterification study like the one carried out by Tharmapuram and Jabarin [60] on PET/PEN blends is required to allow predictions for optimal processing conditions for PEF combined with different (r)PET grades.

### 4.2. Discoloration Considerations

**Figure 6:**
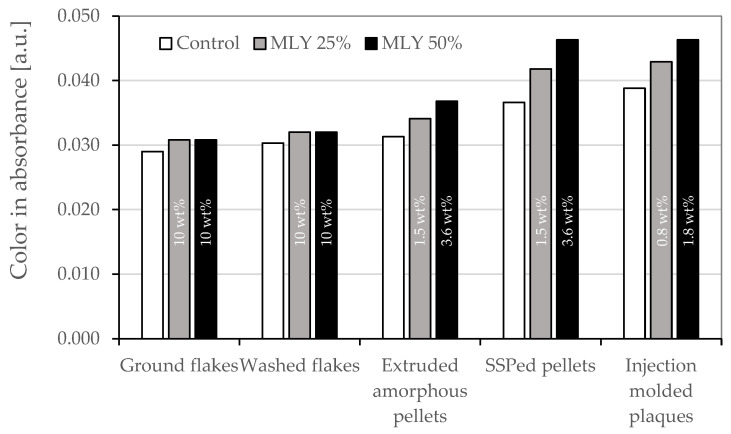
Color in absorbance as calculated from the difference between the absorbance at 400 nm and 780 nm at each stage of the multilayer recycling assessment for the PET reference (“Control”), the PET/PEF multilayer (MLY) at 25% and 50% test concentration. In the bars, the PEF content level as determined using ^1^H-NMR data is denoted (the control has no PEF content).

For each of the recycling assessments presented in this paper, the PEF containing samples showed a higher b* value with an increasing PEF concentration, also shown in Figure 3. For PEF content levels up to 6 wt%, the differences are, however, still within the differences found between different rPET batches; Alvarado Chacon et al. reported, for example, a maximum difference in b* of 3.7 between different rPET batches [13]. The color difference is partially caused by the fact that virgin PEF is currently more yellow than virgin PET. Potentially, this color difference will reduce with the scaling and maturing of FDCA and PEF production technologies. Besides this initial color difference, it is of interest to further explore the source/mechanism for the discoloration of rPET containing PEF. The discoloration of PET when processed/recycled with PLA and MXD6 has been studied before [10,61]. Bandi et al. concluded that the discoloration of the mixture of PET/MXD6 was more severe than the discoloration of the individual components during both melt processing and drying conditions. The AA formed in the PET causes a side reaction with MXD6 to occur, resulting in the formation of colored chromophores [61]. For PEF, no such studies are published, apart from an exploration of the yellowing mechanism of PEF itself [62,63]. Here, the yellowing was linked to intermolecular decarboxylation side reactions, which in turn were promoted to form during melt processing in the presence of Na^+^. Color measurements on dissolved samples in various stages of the recycling assessment process were performed. By measuring in solution, surface or crystallization effects are removed, and the discoloration during each processing step can be analyzed and compared. The absorbance at a wavelength of 400 nm gives an indication of yellowness, whereas the absorbance at a wavelength of 780 nm provides information on the baseline absorbance (includes haze present in the solution). As such, the delta of the absorbance at these wavelengths is a measure of the yellowness of the material. The color evolution of the three tested variables during the multilayer recycling assessment is shown in Figure 6.

The washing step has a minor influence on the color when compared to the extrusion, SSP and injection molding steps. As the recycled PET and PEF content levels change during the injection molding due to the dilution with 50% virgin resin, the influence of the injection molding step is difficult to compare with the previous steps in Figure 6. The contribution of PEF on the color can be estimated with the use of the data of the PET control, the PEF content level and assuming that (i) the contributions of each polymer on the color absorbance data is proportional with its concentration in the solution and (ii) the presence of PEF does not influence the discoloration of PET (and vice versa) during recycling. This leads to the following relation:

(1) $${Color}_{PEF}=\frac{{Color}_{blend}-{Color}_{PET}\cdot \left(100\%-PEF\;content\right)}{PEF\;content}$$

**Figure 7:**
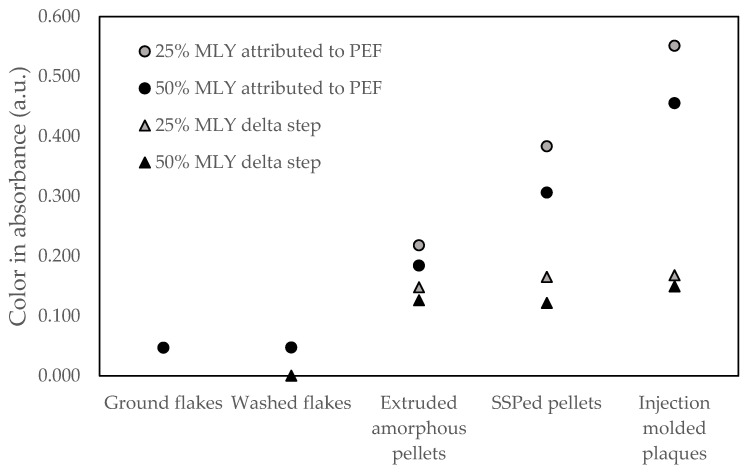
Theoretical color in absorbance of PEF, assuming the color difference between the test variable and the PET control, is attributed to PEF (circles) and its increase for the main steps in the recycling assessment, compared to the previous step (triangles).

The color values attributed to PEF based on these assumptions for the main steps in the recycling assessments are shown in Figure 7. These values are highly dependent on the value of the PEF content level; any measurement inaccuracy will lead to significant deviations. Therefore, it is not surprising that the resulting values for the 25% test concentration are not identical to those of the 50% test concentration. However, the trend is similar, with a comparable contribution to the color for the extrusion, SSP and injection molding step. Interestingly, the estimated PEF color values are about 4 times higher than what has been found for lab and pilot scale recycled PEF after one closed loop in an unpublished internal study. This observation suggests that, alike the outcome of the study of Bandi et al. [61] was a side reaction between MXD6 and the AA in PET, which formed additional colored species, also with the combination of PEF and PET, there might be a reaction mechanism between the two components, leading to additional color formation. Dedicated research on this observation could lead to an important further reduction of the impact of PEF on the color formation during the mechanical recycling of PET. In addition, it would be of interest to explore the color evolution for PEF containing rPET granules when exposed to multiple recycling loops.

### 4.3. Melting Point and Crystallization Rate

**Figure 8:**
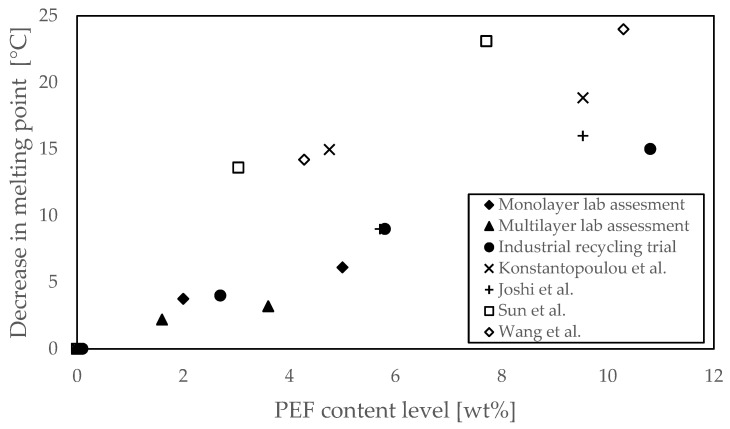
The decrease in melting point compared to the full PET reference in each study, as a function of the PEF content level. The data from the monolayer, multilayer and industrial assessment were completed with literature data. The literature data differs in cooling and subsequent heating rate: Konstantopoulou et al. [42] (x) heating at 20 °C/min on the polymer as is, Joshi et al. [43] (+) heating at 10 °C/min after quench cooling and both Sun et al. [44] (open square) and Wang et al. [45] (open diamond) heating a 10 °C/min after cooling at 10 °C/min.

One of the concerns for recyclers is a considerable drop in melting point, as this could lead to sticking during the post-condensation/decontamination step in the mechanical recycling process for PET. Typically, this step is carried out at temperatures around 210 °C (a range of 190–230 °C is reported by Welle [2]) for mechanical recycling of PET. With a typical melt point of 250–260 °C, the existence of crystals prevents the pellets from softening and sticking [64]. An overview of the melt point reduction as a function of PEF content is shown in Figure 8. The data of the present study are in line with the data on low PEF content levels reported by Joshi et al. [43], with a trend that can be described with a linear relation through the origin. The other datasets found in the literature report higher values for the decline in melting point with an increased PEF content level. It should be noted that the polymer systems in the different datasets differ in molecular weight, degree of randomness, and were exposed to different thermal histories, all of which can be a cause for the differences observed. Another crucial difference between the values from other studies and those of the present study is the absence of IPA as a comonomer in the other studies, where the PET bottle grades used in the present study all contain ~1–2 mol% IPA comonomer. Therefore, the reference melting point of the reference *T*_m_ is already lower than the values reported by the other authors (except for Joshi et al.). The present study is the first to report the melting point for low PEF content levels (<3 wt%), which is the most relevant range for PET recyclers when PEF is introduced in the market. In line with the observed processing behavior during the industrial recycling trial, the reduction of up to 5 °C is not expected to lead to sticking issues.

In Section 3.3.3, the potential benefit of the presence of low PEF content levels in rPET to reduce the crystallization rate of the resulting copolyester to produce thick-walled containers with low haze values was discussed. This general observation of the effect of FDCA moieties incorporated in the rPET on the crystallization speed is consistently confirmed in the literature. Sun et al. [44] have quantified the influence of FDCA and IPA on the crystallization behavior of PET. Data on the supercooling temperature, cold crystallization temperature, peak crystallization temperature, crystallization half-time consistently point to a stronger reduction in crystallization speed for FDCA than IPA when added at the same molar concentration in PET. Sun et al. hypothesize that this stronger effect should be due to both the higher rigidity and polarity of FDCA. The lowest level of PEF content explored by Sun et al. was 3.2 mol%, which is on the higher side of the range relevant for recyclers and the focus for the present study. Further, the crystallization behavior of the copolyester of PET with both FDCA and IPA incorporated in the chains (which is a result of adding PEF in the recycling of bottle-grade PET) has not been described in the literature yet. Therefore, additional research on crystallization of PET and rPET with low PEF content levels will be relevant for recyclers to quantify any risk and explore the potential of PEF entering the PET recycling stream.

## 5. Conclusions

This study demonstrates that the presence of limited amounts of poly(ethylene 2,5-furandicarboxylate) (PEF) in the PET recycling stream does not negatively affect the mechanical recycling process or the performance of recycled PET when present at levels relevant to realistic market introduction scenarios. Both laboratory-scale assessments and industrial-scale validation confirm that PEF contents up to 10 wt% can be processed under standard recycling conditions without adverse effects on regranulation, decontamination, or bottle production. While incorporation of PEF leads to a modest reduction in melting temperature and crystallization rate, these effects remain within an acceptable operational window for the mechanical recycling processes studied and do not induce sticking or processing instabilities. An increase in yellowness with increasing PEF content was observed (though not consistent for all bottles). This discoloration remains comparable to batch-to-batch variations in commercial rPET within the relevant PEF content range and can be effectively mitigated using commercially available toning solutions. The mechanical performance of bottles was found to be unaffected for bottles up to 5 wt% PEF content. Importantly, the reduced crystallization rate imparted by PEF can be beneficial in specific applications, enabling improved clarity in thick-walled or high-rPET-content containers. Overall, the results provide further insights into the effects PET recyclers can anticipate when PEF is introduced in the packaging market on a commercial scale.

## Figures and Tables

**Table 1:** Sample variations evaluated using EPBP route 1 assessment.

	Blend Composition [wt%]		PEF Content [wt%]	
Sample	Reference Flakes	Test Flakes	Extruded Pellets	Plaques
Monolayer control	100	-	-	-
Monolayer 2 wt%	98	2	2	1
Monolayer 5 wt%	95	5	5	2.5
Multilayer control	100	-	-	-
Multilayer 25 wt%	75	25	2.5 ^1^	1.3 ^1^
Multilayer 50 wt%	25	50 t	5 ^1^	2.5 ^1^

^1^ This is the maximum amount, as the elutriation step potentially takes out more PEF barrier layer than PET flakes.

**Table 2:** Overview of extrusion parameters and analytical results of conditions screened.

Sample	Extruder	PEF Content [wt%]	Throughput [kg/h]	*T*_set_ [°C]	*T*_actual_ [°C]	Block Length	Visual Appearance	*T*_g_ [°C] ^1^	*T*_m_ [°C] ^1^
A	conical	10	8	270	278	10	Semi-hazy	80.9	244
B	conical	10	8	290	295	6	Clear	80.0	242
C	conical	10	16	290	292	7	Hazy	80.7	245
D	conical	2	8	290	294	4	Clear	81.0	246
E	conical	5	12	280	286	16	Clear	81.3	246
F	straight	0	5	290		-	Clear	79.6	250
G	straight	10	5	290		3.1	Clear	79.8	240
H	straight	15	5	290		3.6	Clear	78.0	234
I	straight	20	5	290		4	Clear	77.6	230
J	straight	30	5	290		4.5	Clear	79.3	218

^1^ The *T*_g_ was determined from the second heating ramp, and the *T*_m_ was determined from the first heating ramp.

**Table 3:** Overview of analytical results for lab-scale recycling assessment.

			Monolayer			Multilayer		
Sample	Property ^4^	Unit	Control	2 wt%	5 wt%	Control	25 wt%	50 wt%
Bottle ^1^	IV	[dL/g]				0.76	0.78	
	L*	[-]				95.1	94.9	
	a*	[-]				0.0	0.0	
	b*	[-]				0.7	0.8	
	Haze	[%]				0.8	1.4	
Ground flakes ^1^	IV	[dL/g]	0.77	0.74		0.76	0.77	
	L*	[-]	67.1	57.4		62.7	62.9	
	a*	[-]	−0.7	−0.8		−0.5	−0.4	
	b*	[-]	−1.0	0.9		−0.2	−0.6	
Washed flakes ^1^	IV	[dL/g]	0.77	0.74		0.77	0.77	
	Bulk density	[g/mL]	0.32	0.20		0.30	0.19	
	L*	[-]	68.2	63.2		65.7	65.8	
	a*	[-]	−0.9	−1.0		−0.6	−0.5	
	b*	[-]	−0.7	1.5		0.1	−0.4	
Extruded pellets	IV	[dL/g]	0.75	0.75	0.75	0.75	0.75	0.76
	L*	[-]	67.2	66.1	65.5	60.7	60.7	61.0
	a*	[-]	−1.1	−1.3	−0.8	−0.5	−0.4	−0.2
	b*	[-]	2.8	4.3	6.3	2.7	4.0	5.0
SSP’ed pellets	start IV ^2^	[dL/g]				0.74	0.75	0.75
	final IV	[dL/g]	0.84	0.86	0.87	0.87	0.86	0.86
	IV increase rate	[dL/(g·h)]	0.017	0.018	0.018	0.019	0.017	0.018
	L*	[-]	84.4	84.0	81.8	78.8	78.2	77.9
	a*	[-]	−1.6	−1.5	−1.1	−1.0	−0.9	−0.7
	b*	[-]	2.6	3.7	5.1	1.7	2.6	3.8
	*T*_g_ ^3^	[°C]	77.0	80.7	80.9	81.0	81.4	82.0
	*T*_c_ ^3^	[°C]	133	150	156	145	148	153
	*T*_m_	[°C]	245	241	238	244	241	240
	AA content	[ppm]	0.57 ± 0.10	0.48 ± 0.09	0.42 ± 0.04	0.68 ± 0.04	0.55 ± 0.02	0.57 ± 0.03
Plaque	L*	[-]	93.4	93.4	93.0	91.8	91.5	91.1
	a*	[-]	−0.5	−0.7	−0.8	−0.6	−0.7	−0.7
	b*	[-]	3.2	4.2	5.3	4.1	5.2	6.1
	Δb*	[-]	-	1.0	2.1	-	1.1	2.1
	Haze	[%]	2.7	2.4	2.1	3.3	3.4	3.8

^1^ Up to the washed flakes, the values for the PEF-containing samples are on the full PEF flakes (for the 2 wt% and the 5 wt%) or the multilayer bottles/flakes from this bottle (for the 25 wt% and 50 wt%). ^2^ IV of the crystallized pellets, so at the start of the SSP process. ^3^ These transition temperatures were determined from the second heating ramp after quench cooling (instead of a 10 °C/min cooling ramp). ^4^ The color data was measured and supplied by a third-party lab; no information on the amount of repeats or standard deviation is available. Therefore, only the average values are provided. The IV values originate from single-point measurements.

**Table 4:** Preform and bottle properties.

Sample	Property ^1^	Unit	Control	2 wt%	5 wt%
Preform	IV	[dL/g]		0.83	0.82
	L*	[-]		89.67	88.89
	a*	[-]		−0.64	−0.63
	b*	[-]		2.64	3.31
	haze	[%]		19.01	19.42
Bottle	L*	[-]	95.41	95.25	94.83
	a*	[-]	−0.01	−0.01	−0.02
	b*	[-]	0.53	0.77	1.16
	haze	[%]	0.63	0.48	1.26
	burst pressure	[bar]	11.0	11.8 ± 0.2	11.5 ± 0.4
	expansion	[mL]	565	594 ± 57	565 ± 69
	top load	[N]	225	240 ± 21	225 ± 30
	top load elongation	[mm]	2	2.00 ± 0.2	1.73 ± 0.2
	drop test	[passes]	20/20	20/20	20/20
	CO_2_ shelf life	[weeks]		12.5 ± 0.3	13.8 ± 0.3

^1^ The color data was measured and supplied by a third-party lab; no information on the amount of repeats or standard deviation is available, only the average values have been shared.

**Table 5:** Analytical and performance results on recycled PET pellets (and discs and bottles thereof) from a commercial mechanical recycling process with 0 wt%, 2 wt%, 5 wt% and 10 wt% PEF.

			Targeted PEF Content in rPET Sample			
Sample	Property	Unit	0 wt%	2 wt%	5 wt%	10 wt%
SSP’ed pellets	PEF content	[wt%]		2.8	5.9	5.8 ± 4.5 ^6^
3 mm discs ^1^	L*	[-]	70.7 ± 0.1	69.7 ± 0.1	68.9 ± 0.2	68.5 ± 0.1
	a*	[-]	−6.5 ± 0.0	−6.4 ± 0.0	−5.9 ± 0.0	−5.2 ± 0.1
	b*	[-]	9.1 ± 0.6	8.9 ± 0.4	11.9 ± 0.4	15.7 ± 0.7 ± 0.5
	Δb*	[%]	-	−0.2	2.8	6.6
	haze	[%]	18.8 ± 0.8	17.7 ± 0.1	16.8 ± 0.0	16.7 ± 0.1
Bottle ^2^	PEF content ^3^	[wt%]	0.1	2.7	5.8	10.8
	L*	[-]	90.5 ± 0.2	93.4 ± 0.0	93.2 ± 0.0	93.4 ± 0.0
	a*	[-]	−0.6 ± 0.0	−0.7 ± 0.0	−0.9 ± 0.0	−0.8 ± 0.0
	b*	[-]	3.6 ± 0.2	1.6 ± 0.0	2.0 ± 0.0	2.1 ± 0.0
	haze	[%]	6.6 ± 0.4	2.8 ± 0.0	3.2 ± 0.1	3.2 ± 0.1
	*T_m_* ^4^	[°C]	249	245	240	234
	Δ*H_c_* ^4^	[J/g]	37.5	30.1	28.5	8.8
	Crystallinity ^5^	[%]	29	29	27	25
	burst pressure	[bar]	13.2 ± 0.2	11.9 ± 0.1	13.2 ± 0.3	12.3 ± 0.4
	top load	[N]	102 ± 5	81 ± 2	102 ± 6	92 ± 4

^1^ Color measurements on 3 mm discs were performed in triplo. ^2^ Color measurements on the bottle walls were performed in duplo. ^3^ Average PEF content as determined by ^1^H-NMR on two bottles. ^4^ The *T*_m_ was determined from the first heating ramp and the crystallization enthalpy (Δ*H_c_*) from the cooling ramp. Both values were determined from a DSC measurement on a specimen taken from the neck of the bottle. ^5^ The crystallinity was determined from a DSC measurement from a specimen taken from the label area of the wall (the straight section of a bottle). The melt enthalpy of the first heating ramp was converted into the crystallinity using a Δ*H*_m,0_ of 140 J/g for PET [56,57]. ^6^ A large spread in PEF content was measured for the 10 wt% sample. These values are the average and standard deviation for 4 specimens. One specimen was taken from a yellow granule, which had a higher PEF content level (12.4 wt%).

## Data Availability

The original contributions presented in this study are included in the article. Further inquiries can be directed to the corresponding authors.

## Abbreviations

The following abbreviations are used in this manuscript:

AA	Acetaldehyde
APR	Association of Plastic Recyclers
CPBR	Council for PET Bottle Recycling
DSC	Differential scanning calorimetry
EG	Ethylene glycol
EPBP	European PET Bottle Platform
EVOH	Ethylene-vinyl alcohol
FDCA	2,5-furandicarboxylic acid
FEF	FDCA-EG-FDCA moiety
GPC	Gel permeation chromatography
HDPE	High density poly(ethylene)
IPA	Isophthalic acid
IV	Intrinsic Viscosity
MLY	Multilayer
MXD6	Poly(hexamethylenediamine)
NIAS	Non-Intentionally Added Substances
NIR	Near Infra-Red
NMR	Nuclear Magnetic Resonance
PEF	Poly(ethylene 2,5-furandicarboxylate)
PET	Poly(ethylene terephthalate)
PLA	Poly(lactic acid)
PP	Poly(propylene)
PVC	Poly(vinyl chloride)
rPET	Recycled PET
SSP	Solid-state polymerization
TEF	TPA-EG-TPA moiety
TPA	Terephthalic acid
vPET	virgin PET
